# Human Metapneumovirus Epidemiology Among Middle-aged and Older Adults Hospitalized With Acute Respiratory Infection

**DOI:** 10.1093/infdis/jiaf083

**Published:** 2025-07-16

**Authors:** Henrique Pott, Jason J LeBlanc, May ElSherif, Todd F Hatchette, Melissa K Andrew, Shelly A McNeil

**Affiliations:** Canadian Center for Vaccinology, IWK Health, Nova Scotia Health, Dalhousie University, Halifax, Nova Scotia, Canada; Department of Medicine, Universidade Federal de São Carlos, Brazil; Canadian Center for Vaccinology, IWK Health, Nova Scotia Health, Dalhousie University, Halifax, Nova Scotia, Canada; Department of Pathology; Division of Infectious Diseases, Department of Medicine; Canadian Center for Vaccinology, IWK Health, Nova Scotia Health, Dalhousie University, Halifax, Nova Scotia, Canada; Canadian Center for Vaccinology, IWK Health, Nova Scotia Health, Dalhousie University, Halifax, Nova Scotia, Canada; Department of Pathology; Division of Infectious Diseases, Department of Medicine; Canadian Center for Vaccinology, IWK Health, Nova Scotia Health, Dalhousie University, Halifax, Nova Scotia, Canada; Division of Geriatrics, Department of Medicine, Dalhousie University, Halifax, Nova Scotia, Canada; Canadian Center for Vaccinology, IWK Health, Nova Scotia Health, Dalhousie University, Halifax, Nova Scotia, Canada; Division of Infectious Diseases, Department of Medicine

**Keywords:** frailty, human metapneumovirus, older adult, respiratory tract infections

## Abstract

**Background:**

Human metapneumovirus (hMPV) significantly impacts young children, older adults, and those with preexisting conditions. This study examines frailty and clinical outcomes in middle-aged and older adults hospitalized due to hMPV infections.

**Methods:**

Data from the Canadian Immunization Research Network's Serious Outcomes Surveillance Network were analyzed. Included were adults aged ≥50 with lab-confirmed hMPV infection. Severe infection was marked by pneumonia, oxygen need, intensive care unit admission, mechanical ventilation, or death within 30 days. The Frailty Index (FI) categorized individuals as nonfrail (FI < 0.08), prefrail (FI ≥ 0.08, FI < 0.21), and frail (FI ≥ 0.21). Multivariate ridge regression identified factors linked to disease severity.

**Results:**

Among 212 patients (median age 76), 85.4% had severe disease and 61.3% were frail. Frail patients had higher rates of severe disease (80.8% vs. 63.4%), oxygen therapy need (80.8% vs. 63.4%), and longer hospital stays (median 8 vs. 4 days) than patients who were nonfrail. Any cardiac illness (odds ratio [OR], 1.76; 95% confidence interval [CI], 1.02–3.09), congestive heart failure (OR, 1.91; 95% CI, 1.12–3.09), chronic obstructive pulmonary disease (OR, 1.93; 95% CI, 1.14–3.35), and frailty (OR, 1.99; 95% CI, 1.18–3.38) were significantly associated with increased odds of severe disease.

**Conclusions:**

Frailty, assessable with standardized tools, is associated with severe hMPV infections in middle-aged and older adults. Recognizing frailty in clinical management and as a vaccination criterion is necessary as hMPV vaccines become available.

**Clinical Trials Registration:**

NCT01517191 (ClinicalTrials.gov).

Human metapneumovirus (hMPV) is a respiratory disease that easily spreads through contact with contaminated surfaces or bodily fluids [1]. While this virus can affect individuals across all age demographics, it exhibits a disproportionate prevalence among specific populations, including young children, older adults, and those with preexisting health conditions, such as asthma, emphysema, or immunocompromised states [2, 3]. In temperate regions, hMPV infections are most prevalent during late winter and spring, with outbreaks typically lasting approximately 5 months [2, 4]. However, the timing of these outbreaks may vary in tropical regions [4].

In 2021, a systematic review and meta-analysis—based on data from 159 studies conducted worldwide between 2001 and 2019—estimated that there were 14.2 million cases of hMPV infections annually in children aged <5 years [5]. These estimates include >640 000 hospitalizations and 7700 hospital-related deaths. The cases were diagnosed through laboratory confirmation of hMPV-associated acute lower respiratory infections, with the highest hospital admission rates among infants <12 months of age. The mortality rates were notably higher in infants <6 months of age, particularly in low- and lower- and middle-income countries. Although extensive research has focused on young children, individuals of all ages can become infected and experience multiple symptomatic reinfections [6–8]. Data are limited on the burden and severity of hMPV and its outcomes in older adults and adults with comorbidities.

hMPV presents with symptoms similar to those caused by other respiratory viruses, affecting the upper and lower respiratory tract [3, 6, 7]. Currently, there are no specific treatment options available for hMPV infection. Therefore, the primary focus remains on preventing infection and providing supportive care, including oxygen therapy, antipyretics, and intravenous fluids for hydration when necessary [2, 3, 6, 7]. Although no licensed vaccines are currently available for hMPV, research efforts are ongoing, and significant progress has been made toward developing vaccines targeting older adults and individuals with weakened immune systems.

The goal of our study was to address the knowledge gaps regarding the epidemiology of hMPV among middle-aged and older adults and those with comorbidities by exploring the relationship among age, frailty, and clinical outcomes. We focused on (1) describing the demographic and clinical characteristics of patients admitted to the hospital with hMPV; (2) assessing clinical outcomes such as mortality rates, length of hospital stay, and need for mechanical ventilation or intensive care unit (ICU) admission; and (3) identifying factors associated with the severity of hMPV infection, including the level of frailty.

## METHODS

### Data Source

This study utilized data from the Serious Outcomes Surveillance (SOS) Network, part of the Canadian Immunization Research Network (CIRN), to examine the occurrence of acute respiratory illness (ARI) during the 2012–2015 influenza seasons across 7 Canadian provinces: Nova Scotia, New Brunswick, Quebec, Ontario, Manitoba, Alberta, and British Columbia [9–12]. The SOS Network has focused on understanding the impact of influenza and assessing the efficacy of seasonal influenza vaccines. Hospitalized patients aged ≥16 years with ARI—defined as any respiratory condition with or without fever, cough, or unexplained fever—were enrolled in this study. Eligible patients underwent nasopharyngeal swab collection for influenza A/B testing and confirmation by reverse transcription polymerase chain reaction (RT-PCR) as part of standard clinical care. During the study period, nasopharyngeal swabs from individuals aged ≥50 years who met the ARI case definition criteria were tested for influenza A/B and subjected to multiplex RT-PCR to detect hMPV and other common respiratory viruses [10, 11, 13]. Nasopharyngeal swabs were processed at the CIRN SOS Reference Laboratory (Canadian Center for Vaccinology; Halifax, Canada), which used the Seeplex RV15 One-Step ACE Detection assay as its multiplex RT-PCR assay (Seegene, Inc) [13].

### Study Participants

The research ethics board of each participating site approved the study protocol, which included data collection, sample collection, and medical record screening. The study was registered under ClinicalTrials.gov NCT01517191. Additional information about the number of participating sites, including those beyond Halifax, and a detailed description of the ethical procedures followed can be found in the Ethics Approval and Participation Consent section.

The following criteria defined eligible patients for the current study: age ≥50 years, laboratory-confirmed hMPV infection, presentation of ARI symptoms, hospitalization for a minimum of 24 hours, and availability of sociodemographic and clinical characteristics as well as outcome data. The study comprised cases of dual viral infections, all of which were confirmed by RT-PCR. Notably, there are no specific exclusion criteria.

### Data Collection

The CIRN SOS Network follows a standardized procedure for collecting information, which includes gathering detailed data on patients and their medical progress from medical records. In this study, we collected data regarding the age, sex, and living conditions of individuals before hospital admission. We also documented health-related details, such as smoking habits, existing medical conditions (any cardiac illnesses, congestive heart failure [CHF], chronic obstructive pulmonary disease [COPD], diabetes mellitus, chronic kidney disease [CKD], neoplasms, and liver disease), and the need for regular support for activities of daily living. Influenza vaccination status was categorized by whether the patients received the vaccine during the current season or in previous seasons or had never been vaccinated. Additionally, data collection consisted of outcomes such as hospital mortality rates, length of stay, need for noninvasive or mechanical ventilation, and transfer to the ICU.

### Frailty Assessment

Frailty was assessed by a validated deficit accumulation approach [14]. To build the frailty index, we considered various factors, including age-related health conditions, disabilities, and overall function. The index was calculated by adding an individual's health deficits to obtain a total deficit score. This score was then divided by the total number of deficits and transformed into a frailty index from 0 to 1. Frailty was classified into 3 categories: nonfrail (<0.08), prefrail (≥0.08 and <0.21), and frail (≥0.21) [15]. These methods have been used in previous studies by the CIRN SOS Network [10, 12].

### Definition of Disease Severity

Disease severity was assessed in all the participants. Patients who underwent a chest radiograph or computed tomography scan before admission or within 72 hours of admission to the hospital or emergency room were assessed by a treating physician, such as an emergency department physician, radiologist, infectious disease consultant, or internal medicine specialist. These physicians determined whether the chest radiograph or computed tomography findings were consistent with pneumonia.

Lower respiratory tract infection (LRTI) was classified by a composite scoring of symptoms and radiologic findings. Specifically, LRTI was defined as present when the summation of symptoms—including cough, wheezing, sputum production, shortness of breath, and confirmation of pneumonia via chest radiography—reached or exceeded a score of 3.

Given the absence of precise temporal markers for each clinical outcome in the data set, a detailed temporal analysis was rendered infeasible. To address this limitation, a comprehensive strategy for defining case severity was implemented. “Severe” hMPV infection was determined by the presence of any LRTI identified through the specified composite criteria, supplemented by additional clinical indicators. These included the requirement for supplemental oxygen during hospitalization, admission to the ICU, necessity for mechanical ventilation, and/or death from any cause within 30 days of hospital admission. Patients who did not meet these conditions were categorized as “nonsevere,” allowing for a more structured assessment of clinical outcomes based on a combination of symptomatology and radiologic evidence, despite the lack of temporal information to track the progression of each outcome.

### Hospital Outcomes

Participants were monitored from admission until discharge or death. Our assessment of disease burden included a comparison of various in-hospital outcomes, focusing on length of stay, 7- and 30-day mortality rates, and complication rates. Evaluated complications included the need for mechanical ventilation and ICU admission within 30 days. These outcomes were analyzed with a focus on stratifying patients according to frailty levels to better understand the relationship between frailty and severity of hMPV infection.

### Statistical Analysis

Quantitative data are expressed as median and IQR, following an assessment of normality with the Shapiro-Wilk test. Categorical data are presented as absolute numbers accompanied by their relative frequencies in parentheses. The Brown-Mood median test was used to compare quantitative data, while comparisons of categorical data were conducted by Fisher exact test.

To analyze the factors associated with the severity of hMPV infection, ridge regression was employed to address challenges such as overfitting, multicollinearity, and unstable coefficient estimates, which are often observed in traditional logistic regression [16]. The dependent variable was disease severity, while the independent variables were age, sex, history of cardiac issues, CHF, COPD, diabetes, renal disease, cancer, and frailty index category, selected for their clinical relevance to the disease [17, 18]. Odds ratio (OR) and 95% CI were estimated by a bootstrapping procedure with 1000 resamples, ensuring robust and precise measurements of association.

Statistical significance was assessed by a 2-sided *P* < .05. All analyses were performed with R (version 4.4.0, “Puppy Cup” release) and RStudio IDE (version 2024.04.0+735, “Chocolate Cosmos” release).

## RESULTS

In our investigation, 212 hMPV infections were identified in patients with ARI. Of these, 85.4% (n = 181) were categorized as severe. Among the severe cases, 54.0% (n = 114) were attributed to LRTI, and within these cases, 26.3% (n = 30) presented with pneumonia, highlighting the significant severity within this cohort.

### Sociodemographic and Clinical Characteristics

Table 1 presents the sociodemographic and clinical characteristics of the participants. The median age of the participants with hMPV was 76 years (IQR, 65–86): 24.5% were aged 50 to 64 years; 20.8%, 65 to 74; 26.9%, 75 to 84; and 27.8%, ≥85. Females represented 56.6% of the population. Regarding living arrangements, 82.1% of respondents resided in private community dwellings, 15.1% in assisted living or long-term care facilities, 1.9% in community group homes, and 0.5% were homeless or had undetermined living situations. Regarding smoking history, 45.8% were former smokers and 12.7% were current smokers. Medical histories indicated that 48.1% of the participants had underlying cardiac disease, 17.0% CHF, 27.8% COPD, 26.4% diabetes, 11.8% CKD, and 24.1% cancer. The majority (59.4%) of the patients presented with 1 or 2 comorbidities. Furthermore, 56.6% received regular assistance with activities of daily living before admission, and 71.7% were vaccinated against influenza during the enrollment season. Most patients (61.3%) were frail with a median frailty index of 0.26 (IQR, 0.14–0.34).

**Table 1.** Sociodemographic and Clinical Characteristics at Baseline: An Overview

Variable	No. (%) or Median (IQR)
No. of patients	212
Age, y	76 (65–86)
Age group, y	
50–64	52 (24.5)
65–74	44 (20.8)
75–84	57 (26.9)
≥85	59 (27.8)
Sex	
Female	120 (56.6)
Male	92 (43.4)
Baseline living	
Community private house	174 (82.1)
Assisted living/long-term care facility	32 (15.1)
Community group home	4 (1.9)
Shelter/homeless	1 (0.5)
Unknown	1 (0.5)
Smoking status	
Never smoked	87 (41.0)
Former smoker	97 (45.8)
Current smoker	27 (12.7)
Unknown	1 (0.5)
Comorbidities	
Any cardiac illnesses	102 (48.1)
Congestive heart failure	36 (17.0)
Chronic obstructive pulmonary disease	59 (27.8)
Diabetes mellitus	56 (26.4)
Chronic kidney disease	25 (11.8)
Neoplasms	51 (24.1)
Liver disease	5 (2.4)
No. of comorbidities	
0	40 (18.9)
1 or 2	126 (59.4)
≥3	46 (21.7)
Baseline support: require regular support for activities of daily living	119 (56.1)
Influenza vaccination status	
Never vaccinated	50 (23.6)
Current season vaccination	152 (71.7)
Vaccination in prior seasons only	10 (4.7)
Season of enrollment	
2012–2013	65 (30.7)
2013–2014	117 (55.2)
2014–2015	30 (14.2)
Frailty assessment	
Frailty index	0.26 (0.14–0.34)
Nonfrail: <0.08	25 (11.8)
Prefrail: ≥0.08 and <0.21	57 (26.9)
Frail: ≥0.21	130 (61.3)
Coinfection	
hMPV-influenza	16 (7.5)
hMPV-RSV	2 (0.9)

Abbreviations: hMPV, human metapneumovirus; RSV, respiratory syncytial virus.

### Disease Severity

Significant differences were identified between the nonsevere and severe groups in various demographic and clinical characteristics. Table 2 provides a stratified presentation of the findings. Regarding age distribution, severe cases were more prevalent in the groups aged 75 to 84 years (28.7%) and ≥85 years (28.2%), whereas the nonsevere group exhibited a higher proportion of individuals aged 50 to 64 years (32.3% vs 23.2%), although this difference was not statistically significant.

**Table 2.** Clinical Characteristics According to Disease Severity

	No. (%) or Median (IQR)			
Variable	Overall	Nonsevere	Severe	*P* Value
No. of patients	212	31	181	…
Age group, y				.399
50–64	52 (24.5)	10 (32.3)	42 (23.2)	
65–74	44 (20.8)	8 (25.8)	36 (19.9)	
75–84	57 (26.9)	5 (16.1)	52 (28.7)	
≥85	59 (27.8)	8 (25.8)	51 (28.2)	
Sex				.232
Female	120 (56.6)	14 (45.2)	106 (58.6)	
Male	92 (43.4)	17 (54.8)	75 (41.4)	
Smoking status				.058
Never smoked	87 (41.0)	11 (35.5)	76 (42.0)	
Former smoker	97 (45.8)	13 (41.9)	84 (46.4)	
Current smoker	27 (12.7)	6 (19.4)	21 (11.6)	
Unknown	1 (0.5)	1 (3.2)	0 (0.0)	
Comorbidities				
Any cardiac illnesses	102 (48.1)	8 (25.8)	94 (51.9)	.012
Congestive heart failure	36 (17.0)	1 (3.2)	35 (19.3)	.051
Chronic obstructive pulmonary disease	59 (27.8)	4 (12.9)	55 (30.4)	.074
Diabetes mellitus	56 (26.4)	5 (16.1)	51 (28.2)	.235
Chronic kidney disease	25 (11.8)	5 (16.1)	20 (11.0)	.610
Neoplasms	51 (24.1)	9 (29.0)	42 (23.2)	.635
Liver disease	5 (2.4)	1 (3.2)	4 (2.2)	>.99
Influenza vaccination status				>.99
Unvaccinated in the current season	60 (28.3)	9 (29.0)	51 (28.2)	
Current season vaccination	152 (71.7)	22 (71.0)	130 (71.8)	
Frailty assessment				
Frailty index	0.26 (0.14–0.34)	0.14 (0.09–0.26)	0.27 (0.15–0.36)	<.001
Nonfrail: <0.08	25 (11.8)	7 (22.6)	18 (9.9)	.005
Prefrail: ≥0.08 and <0.21	57 (26.9)	13 (41.9)	44 (24.3)	
Frail: ≥0.21	130 (61.3)	11 (35.5)	119 (65.7)	
Coinfections				.753
hMPV-influenza	16 (7.5)	3 (9.7)	13 (7.2)	
hMPV-RSV	2 (0.9)	0 (0.0)	2 (1.1)	
Chest radiograph/CT scan				<.001
Normal	23 (10.8)	7 (22.6)	16 (8.8)	
Altered				
No pneumonia	156 (73.6)	22 (71.0)	134 (74.0)	
Pneumonia	30 (14.2)	0 (0.0)	30 (16.6)	
Not available	3 (1.4)	2 (6.5)	1 (0.6)	
Symptoms				
Cough	183 (86.3)	20 (64.5)	163 (90.1)	<.001
Wheezing	75 (35.4)	3 (9.7)	72 (39.8)	.002
Sputum production	109 (51.4)	5 (16.1)	104 (57.5)	<.001
Shortness of breath	161 (75.9)	11 (35.5)	150 (82.9)	<.001

Abbreviations: CT, computed tomography; hMPV, human metapneumovirus; RSV, respiratory syncytial virus.

**Figure 1.**
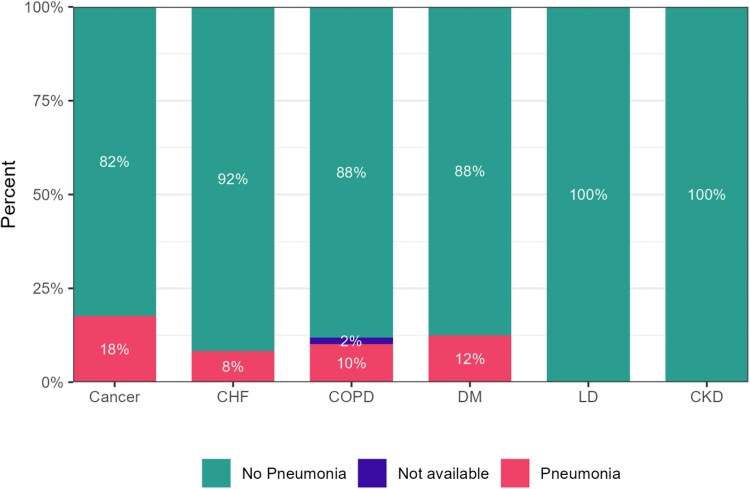
Percentage of patients diagnosed with pneumonia due to human metapneumovirus infection, categorized by underlying comorbidities. Abbreviations: CHF, congestive heart failure; CKD, chronic kidney disease; COPD, chronic obstructive pulmonary disease; DM, diabetes mellitus; LD, liver disease.

When comorbidities were examined, a significantly higher percentage of patients with severe disease had underlying cardiac conditions (51.9% in the severe group vs 25.8% in the nonsevere group, *P* = .013), and CHF was more prevalent in the severe group (19.3% vs 3.2%, *P* = .051). The median frailty index was also higher in the severe group than in the nonsevere group (0.27 vs 0.14, *P* < .001), and frailty was more frequently associated with severe disease status (*P* < .001). Radiologic findings indicated that 16.6% of the patients with severe disease exhibited pneumonia, whereas no such cases were observed in the nonsevere group (Figure 1). Additionally, patients with severe disease showed higher rates of cough, sputum production, and dyspnea, emphasizing symptomatic differences between the groups.

### Hospital Outcome Analysis by Frailty

Table 3 provides a detailed account of the clinical outcomes in the study cohort, stratified according to frailty status. The data indicate that 85.4% of patients were classified as having severe disease, with this proportion being higher in individuals who were frail (91.5%) vs nonfrail (75.6%). Radiologic assessment revealed that 14.2% of the participants were diagnosed with pneumonia. The prevalence of pneumonia was slightly higher in patients who were frail (15.4%) than nonfrail (12.2%).

**Table 3.** Clinical Outcomes According to Frailty

	No. (%) or Median (IQR)			
Parameter	Overall	Nonfrail (FI < 0.21)	Frail (FI ≥ 0.21)	*P* Value
No. of patients	212	82	130	…
Disease severity				.002
Nonsevere	31 (14.6)	20 (24.4)	11 (8.5)	
Severe	181 (85.4)	62 (75.6)	119 (91.5)	
Chest radiograph/CT scan				.066
Normal	23 (10.8)	12 (14.6)	11 (8.5)	
Altered				
No pneumonia	156 (73.6)	57 (69.5)	99 (76.2)	
Pneumonia	30 (14.2)	10 (12.2)	20 (15.4)	
Not available	3 (1.4)	3 (3.7)	0 (0.0)	
Mortality				
7 d in hospital	1 (0.5)	0 (0.0)	1 (0.8)	>.99
30 d in hospital	5 (2.4)	0 (0.0)	5 (3.8)	.182
Length of stay: days of hospitalization	6 (4–11)	4 (3–9)	8 (4–13)	.001
Complication				
Oxygen therapy during admission	157 (74.1)	52 (63.4)	105 (80.8)	.008
Mechanical ventilation	13 (6.1)	7 (8.5)	6 (4.6)	.387
Admission to the ICU				
7 d in hospital	21 (9.9)	9 (11.0)	12 (9.2)	.858
30 d in hospital	23 (10.8)	10 (12.2)	13 (10.0)	.784

Abbreviations: CT, computed tomography; FI, frailty index; ICU, intensive care unit.

The in-hospital mortality rate was 2.4% (5/212). All deaths within 7 days involved frail cases, accounting for 0.5% of the cohort. The 30-day in-hospital mortality rate of patients who were frail was 3.8%, with no fatalities in the nonfrail group (*P* = .182). Regarding hospitalization duration, the median length of stay was longer for the frail group at 8 days (IQR, 4–13) than for the nonfrail group at 4 days (IQR, 3–9; *P* = .001). Oxygen therapy was more frequently required by patients who were frail (80.8%) than by their counterparts who were not frail (63.4%). Mechanical ventilation was necessary in 6.1% of the cases overall, with no significant differences between the frail and nonfrail groups. The ICU admission rates at 7 and 30 days were 9.9% and 10.8%, respectively, with no significant variation observed between the groups.

### Factors Associated With Disease Severity

A ridge regression model was used to assess disease severity based on factors such as age, sex, cardiac history, CHF, COPD, diabetes, renal disease, cancer, and frailty (Table 4). It found frailty to be a significant predictor of severe disease (OR, 1.99; 95% CI, 1.18–3.38), cardiac disease (OR, 1.76; 95% CI, 1.02–3.09), CHF (OR, 1.91; 95% CI, 1.12–3.09), and COPD (OR, 1.93; 95% CI, 1.14–3.35). Other covariates—including sex, age, and comorbidities (eg, diabetes, CKD, and cancer)—exhibited varying associations with disease severity, albeit without statistical significance. Liver disease was excluded from the model due to insufficient cases.

**Table 4.** Results of Ridge Regression Model Identifying Factors Associated With Disease Severity

Parameter	Odds Ratio (95% CI)
Age, y	0.99 (.98–1.02)
Sex	
Female	1 [Reference]
Male	0.65 (.41–1.15)
Comorbidity	
Any cardiac illnesses	1.76 (1.02–3.09)
Congestive heart failure	1.91 (1.12–3.09)
Chronic obstructive pulmonary disease	1.93 (1.14–3.35)
Diabetes mellitus	1.49 (.83–2.69)
Chronic kidney disease	0.49 (.22–1.25)
Neoplasms	0.90 (.51–1.71)
Frailty assessment	
Nonfrail	1 [Reference]
Frail	1.99 (1.18–3.38)

Liver disease was excluded from the model due to insufficient cases.

## DISCUSSION

This study examined the sociodemographic and clinical characteristics of middle-aged and older adults hospitalized with hMPV infection, with a focus on frailty and disease severity. We found that being older and female, living with underlying comorbidities, and frailty were associated with hMPV infection and severe illness.

The demographic profile, with a median age of 76 years and mostly female patients (56.6%), is consistent with previous research on hMPV infections among older adults [19–21]. This consistency validates the study and underscores the necessity for targeted prevention and management strategies for this susceptible group. The observed age and sex distribution could be attributed to several factors, such as immunosenescence, frailty levels, and potential sex differences in health care–seeking behavior [12]. While the existing literature lacks a focus on the higher prevalence of hMPV incidence in females, it is noteworthy that the predominance of female patients could be due to a longer life expectancy or, potentially, as is most often the case, greater frailty in females than males [22]. Older age has consistently been identified as a risk factor for respiratory viral infections in adults and is predictive of adverse clinical outcomes [23, 24].

The study cohort exhibited a high prevalence of comorbidities, particularly cardiac illnesses (48.1%), COPD (27.8%), diabetes (26.4%), and neoplasms (24.1%), highlighting the complex health scenarios faced by older adults with hMPV infections. Notably, cases of pneumonia were frequently observed, underscoring the potential clinical severity of concurrent respiratory infections. These findings align with previous research identifying chronic medical conditions and respiratory infections such as pneumonia as significant risk factors for severe respiratory viral infections in older populations [25]. The association between any cardiac disease, CHF, or COPD and severe hMPV infection highlights the need for targeted interventions. This link may stem from the increased vulnerability of patients with these comorbidities, who are more likely to experience exacerbations of their underlying conditions or have reduced tolerance in the face of such exacerbations.

Our analysis did not show a statistically significant association between CKD and disease severity (OR, 0.49; 95% CI, 0.22–1.25). This contrasts with previous studies, which suggested an increased risk among patients with CKD and respiratory viral infections [25, 26]. The adequacy of the sample size may limit the ability to make a firm assessment of this association, as a smaller cohort can increase the likelihood of statistical anomalies. Additionally, several unmeasured confounding factors could influence this distribution, leading to a potential spurious association. These factors may include differences in health care access and variations in admission thresholds, particularly to support dialysis, or distinct clinical presentations in patients with CKD who might experience more nonrespiratory manifestations of hMPV. Consequently, further investigations are needed to clarify the potential protective mechanisms or confounding factors underlying this association and to validate these findings in larger, more diverse study populations.

Frailty was associated with disease severity, with patients who were frail exhibiting a significantly higher OR (1.99; 95% CI, 1.18–3.38). Interestingly, age was not significantly associated with disease severity, underscoring the importance of considering frailty as a more pertinent parameter than chronologic age. This finding aligns with and enriches the growing body of evidence advocating the inclusion of frailty assessments in the daily emergency care routine [27, 28]. Previous studies have consistently demonstrated that frailty, rather than age alone, substantially influences various health outcomes in older adults, including the progression and severity of respiratory infections. As the traditional reliance on age as a sole indicator of health vulnerability is reevaluated, frailty offers a comprehensive measure that accounts for the cumulative decline in physiologic reserves, providing a more accurate risk stratification and targeted management approach. Frailty can be assessed by standard methods such as the frailty index, which encompasses a broad range of accumulated health deficits [29]. Another widely recognized approach is the Clinical Frailty Scale, which evaluates an individual's overall level of fitness and frailty based on clinical judgment and a short questionnaire [30]. Implementing these standardized assessments facilitates objective and reliable measurement of frailty, thereby guiding appropriate interventions. Ultimately, this shift toward recognizing frailty over age enhances our ability to personalize interventions and improve clinical outcomes for older adults, emphasizing the nuanced understanding required in health care.

The clinical outcomes observed in this study offer valuable insights into the trajectory of hMPV infection in older adults. Notably, the relatively low mortality rates (0.5% at 7 days and 2.4% at 30 days) are encouraging as compared, for example, with a mortality rate of 6.1% from RSV in the same surveillance network [11], suggesting that the prognosis of older adults with hMPV infection can be relatively favorable with appropriate management. Despite this somewhat positive outlook, the substantial proportion of patients requiring oxygen therapy (74.1%), a median hospital stay of 6 days, and the observed 30-day mortality rate of 2.4% highlight the considerable burden that hMPV infections impose on health care resources. This extended hospitalization strains health care facilities and increases the risk of additional complications not measured in this study, including hospital-acquired infections, alterations in patients’ functionality and frailty levels, and potential readmissions [31].

We acknowledge the limitations inherent in the observational design of this study, which constrain its capacity to establish causal inferences. By focusing on hospitalized patients diagnosed with hMPV, we provide a detailed examination of severe cases but limit our ability to ascertain the incidence of severe hMPV disease in the general population. This focus, while yielding insights into the progression of severe cases, requires careful consideration when attempting to generalize the findings to all hMPV infections. Future research should incorporate nonhospitalized cases to provide a more comprehensive understanding of the disease incidence. Nevertheless, the multicenter design of our study enhances the generalizability of our findings across diverse settings and populations, offering valuable insights into hMPV infections in middle-aged and older adults. Despite this advantage, the study faced challenges, such as a relatively small sample size, especially within the nonsevere group, which may have affected the statistical power to discern certain associations. This limitation underscores the need for caution when interpreting the findings, as smaller samples can lead to reduced reliability and increased outcome variability. Additionally, the variability and imbalance across severity categories prevented us from employing an ordinal regression model to fully capture the spectrum of clinical severity, thus limiting our ability to explore nuanced grades between outcomes. Variability in clinical practices and patient management across centers could also introduce heterogeneity, potentially influencing the consistency of the results. In addition, this surveillance was conducted during 3 influenza seasons, and hMPV circulation and illness outside the influenza season would thus have been missed. Despite these limitations, this study provides valuable groundwork for further research, highlighting areas where future investigations could incorporate larger, more diverse cohorts and longitudinal designs to better establish causality and refine our understanding of the disease dynamics in this population.

In conclusion, this study provides valuable insights into the characteristics and outcomes of hMPV infection in middle-aged and older adults, highlighting the role of frailty as a risk factor for severe illnesses. These findings underscore the importance of comprehensive geriatric assessments, including frailty evaluations, in managing respiratory viral infections in this population. Future research should aim to develop and validate risk prediction models incorporating frailty measures and specific comorbidities to guide clinical decision making, improve outcomes, and inform vaccine recommendations for older adults with hMPV infections.
